# Mio-Pliocene Faunal Exchanges and African Biogeography: The Record of Fossil Bovids

**DOI:** 10.1371/journal.pone.0016688

**Published:** 2011-02-16

**Authors:** Faysal Bibi

**Affiliations:** Institut de Paléoprimatologie et Paléontologie Humaine: Evolution et Paléoenvironnements CNRS UMR 6046, Université de Poitiers, Poitiers, France; University of Arkansas, United States of America

## Abstract

The development of the Ethiopian biogeographic realm since the late Miocene is here explored with the presentation and review of fossil evidence from eastern Africa. *Prostrepsiceros* cf. *vinayaki* and an unknown species of possible caprin affinity are described from the hominid-bearing Asa Koma and Kuseralee Members (∼5.7 and ∼5.2 Ma) of the Middle Awash, Ethiopia. The Middle Awash *Prostrepsiceros* cf. *vinayaki* constitutes the first record of this taxon from Africa, previously known from the Siwaliks and Arabia. The possible caprin joins a number of isolated records of caprin or caprin-like taxa recorded, but poorly understood, from the late Neogene of Africa. The identification of these two taxa from the Middle Awash prompts an overdue review of fossil bovids from the sub-Saharan African record that demonstrate Eurasian affinities, including the reduncin *Kobus porrecticornis*, and species of *Tragoportax*. The fossil bovid record provides evidence for greater biological continuity between Africa and Eurasia in the late Miocene and earliest Pliocene than is found later in time. In contrast, the early Pliocene (after 5 Ma) saw the loss of any significant proportions of Eurasian-related taxa, and the continental dominance of African-endemic taxa and lineages, a pattern that continues today.

## Introduction

Wallace [1], following Sclater [2], classified the majority of Africa and Arabia into a single ‘Ethiopian’ biogeographic realm, extending from the Tropic of Cancer southwards to the Cape and Madagascar. Wallace was struck by both the high number of animal groups endemic to this area as well as the absence from it of many widespread Eurasian taxa. He wrote (p.253):

*"The great speciality indicated by [the Ethiopian realm's] numerous peculiar families and genera, is still farther increased by the absence of certain groups dominant in the Old-World continent, an absence which we can only account for by the persistence, through long epochs, of barriers isolating the greater part of Africa from the rest of the world.”*

**Figure 1:**
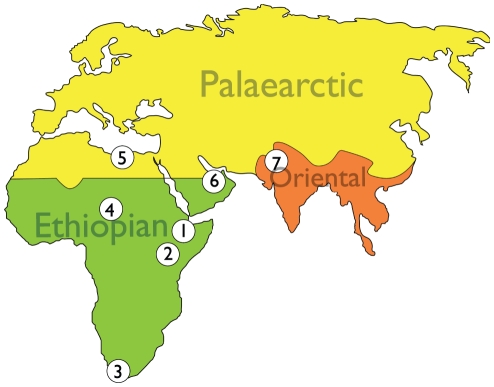
Map showing the boundaries of modern day biogeographic realms and the locations of sites mentioned in the text. 1, Middle Awash; 2, Lothagam, Lukeino, Mpesida, Namurungule; 3, Langebaanweg; 4,Toros-Menalla; 5, Sahabi; 6, Baynunah; 7, Siwaliks.

More than 130 years on, the biogeographic scheme of Sclater and Wallace continues to form a basis for continental-scale geographic comparison of mammalian communities (Fig. 1). Any observer of modern Africa can quickly recognize the stark ecological boundary delimited by the Sahara Desert, with the vast diversity of African-endemic taxa restricted to regions to its south. With almost no African fossil record to consult, scientists of the 19th and early 20th centuries could only speculate on the age or historical development of this continent's biogeography. In contrast, the last 100 years of paleontological exploration have provided a wealth of information that allows for an investigation into the developmental history of African endemism as a whole, and the Ethiopian biogeographic realm in particular. Wallace's proposal of “long epochs” of isolating barriers can now be more precisely formulated and addressed.

I here investigate the development of African and Ethiopian realm endemism, relying primarily on the fossil record of Bovidae (antelopes, oxen, and kin). I begin by describing three fossil bovid specimens from the faunas of the Asa Koma Member of the Adu-Asa Formation (ASKM) and Kuseralee Member of the Sagantole Formation (KUSM) of the Middle Awash, Ethiopia, dated to 5.77–5.54 Ma and ∼5.2 Ma, respectively [3]. These specimens represent two new additions to the faunal lists from those assemblages, which include the hominid *Ardipithecus kadabba* [4]. I follow with a review of the development of African biogeographic endemism over the last 8 Ma as informed by the presence of bovids of Eurasian affinities in the sub-Saharan African fossil record, outlining the history of the Ethiopian biogeographic realm since the late Miocene.

## Results

### Systematic Paleontology Antilopini Gray 1821, *Prostrepsiceros* Major 1891, *Prostrepsiceros* cf. *vinayaki* (Pilgrim 1939)

#### Revised Diagnosis

A bovid of medium to small size characterized by horn cores that arise from above the orbit with moderate inclination and basal divergence, torsion that is anticlockwise in the right horn core and relatively helical, very strong mediolateral compression with an oval basal cross-section and flat antero-medial surface, a prominent anterior keel that originates anteromedially, and a posterior keel that is variable in expression. Shallow postcornual fossa present, supraorbital foramina small, pear-shaped, single or multiple.

#### Holotype

GSI B799, left horn core from the locality of Nila, Dhok Pathan Formation, Siwaliks ([5]: pl. I fig. 10).

#### Referred specimens

**Figure 2:**
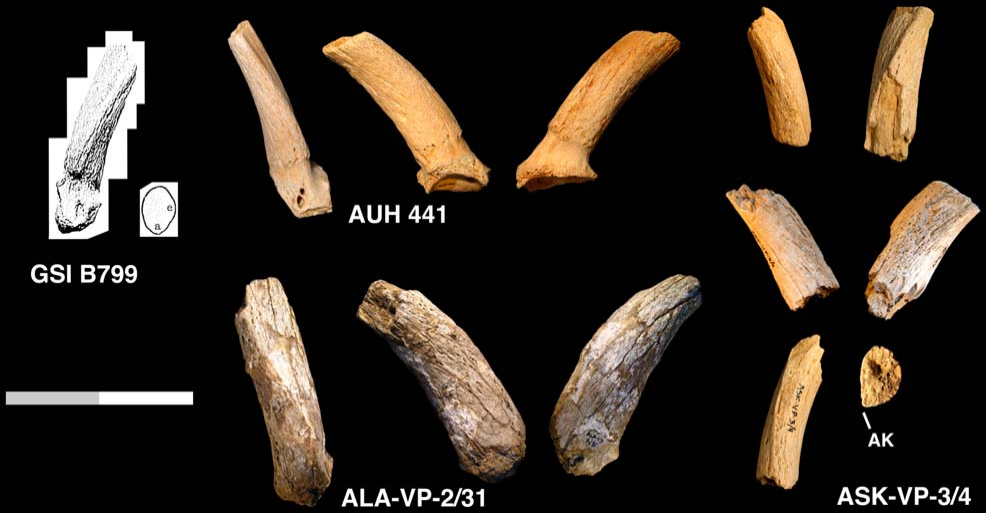
GSI B799, holotype left horn core of *Prostrepsiceros vinayaki* from the Dhok Pathan Formation, Siwaliks (reproduced from [5]). AUH 441, right horn core of *P.* aff. *vinayaki* from the Baynunah Formation (U.A.E.) in anterior, lateral, and medial views. ALA-VP-2/31, right horn core of *P.* cf. *vinayaki* in anterior, lateral, and medial views. ASK-VP-3/4, right and left horn core fragments of *P.* cf. *vinayaki*, in anterior views (top row), lateral views (middle row), right horn core fragment in posterior view, and view of distal break of left horn core fragment with anterior keel position marked AK. Scale bar equals 10 cm total (approximative for GSI B799).

ASK-VP-3/4, proximal portions of right & left horn cores. ALA-VP-2/31, base of right horn core (Fig. 2).

#### Age

Sites ASK-VP-3 and ALA-VP-2 are situated within the Asa Koma Member of the Adu-Asa Formation, and are constrained to between 5.77 and 5.54 Ma, the respective ages of the LABT tuff and the MA95-7 basaltic lava [6].

#### Description

ASK-VP-3/4: right and left horn core fragments: left is a basal portion preserving a bit of the pedicel-horn core boundary, right is from just above the horn core base. Both the anterior and posterior keels are strong, the medial surface is quite flattened and the lateral surface rounded. Transverse and antero-posterior diameters (DTxDAP) at just above the horn core base in the left is 23.7×31.8, and at the proximal break in the right 20.6×29.5 mm. ALA-VP-2/31: proximal right horn core with part of the frontal, somewhat weathered, larger in size than ASK-VP-3/4. Anterior keel is prominent and the posterior keel weak. Medial surface is quite flat and the lateral face rounded such that the basal cross-section is an asymmetric oval. A small foramen is present at the posterolateral side of the horn core-pedicel junction. In medial view, the break through the frontal shows no indication of frontal sinuses. Basal DTxDAP is 26.6×38.5 mm.

#### Comparisons

The ASKM specimens can be differentiated from *Sivoreas eremita* by horn cores that are much more mediolaterally-compressed, with cross-section more asymmetrical, and weaker torsion that is wider to the torsion axis (more helical), with significant lateral divergence of the horn cores above the base (torsion is tight and the horn core relatively straight in *S. eremita*). They differ from *Prostrepsiceros libycus* in the presence of a strong anterior keel, stronger mediolateral torsion, a medial surface that is flatter than the lateral one, and the absence of grooves running along the anterior and posterolateral faces. In all these characters, the ASKM specimens are a good match for *Prostrepsiceros vinayaki*.

*Prostrepsiceros vinayaki* is otherwise represented by only a few horn core specimens. These are the holotype [5], two other horn cores from the Siwaliks [7], and a horn core (and three postcranial specimens) from the Baynunah Formation referred to *Prostrepsiceros* aff. *vinayaki* [8]. Additional material attributable to *Prostrepsiceros vinayaki* or *P.* cf. *vinayaki* and awaiting description is known from the Siwaliks (A. Gentry and J. Barry pers. comm.), Molayan in Afghanistan [9], and Marageh, Iran [10].

**Figure 3:**
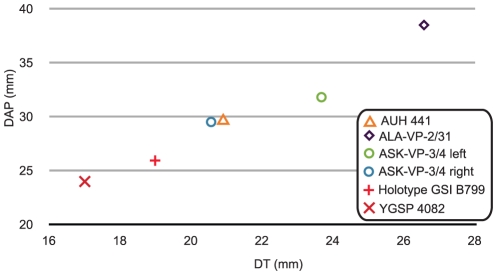
Plot of basal horn core anteroposterior and transverse diameters in *Prostrepsiceros vinayaki* (except in ASK-VP-3/4 where measurements are from just above the base). Data from Pilgrim [5], Thomas (my estimation from pl. III fig. 7 in [7]), and Gentry [8].

The paucity and poor representation of material of *P. vinayaki* limits comparisons. The two Awash specimens, particularly ALA-VP-2/31, are larger than all three previously known horn cores for which measurements are available (Fig. 3). Gentry [8] reports the Baynunah horn core to have a flatter latter than medial surface, though I have found the surface just medial to the anterior keel to be the flattest part of the horn core. A difference amongst the known specimens of this taxon concerns the prominence of the posterior keel, which is reported as absent in the holotype, weak in the Baynunah specimen, weak to prominent in the Middle Awash specimens, or even more prominent than the anterior keel in other Siwaliks specimens. Otherwise, the Middle Awash, Siwaliks, and Baynunah horn cores all bear a combination of horn core characters that is unique and characteristic, though more and better material will have to be found and described to confidently determine whether this sample represents one or more species.

#### Notes

**Figure 4:**
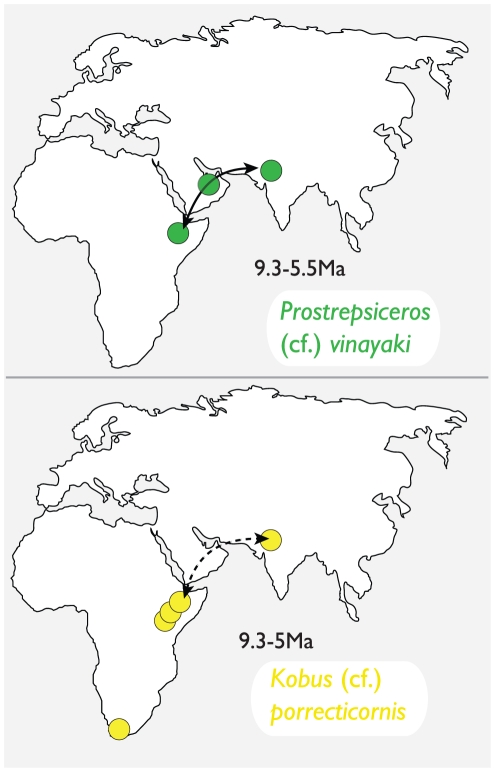
Maps plotting records of *Prostrepsiceros vinayaki* (including *P.* cf. *vinayaki*) and *Kobus porrecticornis* (including *K.* cf. *porrecticornis*), highlighting links between southern Asia, Arabia, and East Africa in the late Miocene.

The assignment of ASK-VP-3/4 and ALA-VP-2/31 to *Prostrepsiceros* cf. *vinayaki* constitutes the first record of this taxon in Africa (Fig. 4). *Prostrepsiceros vinayaki* is recorded in the Siwaliks to span 9.3–7.9 Ma [11], both the Molayan and the Baynunah faunas are between 8 and 6 Ma [12], [13], [14], [15], and the new Middle Awash occurrences are between 5.77 and 5.54 Ma [16]. These specimens then span around 4myr, which would be atypical for the duration of a single bovid species. Regardless, the morphological congruence of the Awash, Baynunah, Siwaliks, and Molayan records is here taken to be of phylogenetic (and resulting biogeographic) significance.

Kostopoulos [9] presented additional information on the cranial morphology of *P. vinayaki*, presumably from the unpublished Molayan material. In a systematic review, Kostopoulos found the closest affinities to *P. vinayaki* in the Greco-Anatolian *P. vallesiensis*, stating that “these two species seem to comprise a morphological continuum” (p889). Recent Siwaliks data [11] indicate that *P. vallesiensis* and *P. vinayaki* have a similar first appearance datum, and the phylogenetic relationships proposed by Kostopoulos [9] are of interest in that they suggest the most recent common ancestor of these two species existed in the early late Miocene and probably inhabited the Greco-Iranian region.

Other reported occurrences of *Prostrepsiceros* from Africa come from Oued el Atteuch (Algeria) and Sahabi (Libya). The Oued el Atteuch record [17] consists of a tooth and a horn core fragment which, to my knowledge, have not been described or figured. The Sahabi *Prostrepsiceros libycus* [18] differs markedly from *P. vinayaki* and has recently been reassigned to *Dytikodorcas* [19]. ALA-VP-2/31 was previously referred to *Aepyceros* cf. *premelampus* by Haile Selassie et al. [20]. The only other ASKM specimen that these authors assigned to *Aepyceros* is ALA-VP-1/5, which in fact bears a triangular section and pronounced torsion and is better assigned to *Tragelaphus moroitu*. These reassignments now restrict the occurrence of *Aepyceros* in the Mio-Pliocene Middle Awash assemblages to the younger Kuseralee deposits, with implications discussed below.

### Incertae Sedis cf. Caprini Gray 1821

#### Referred specimens

**Figure 5:**
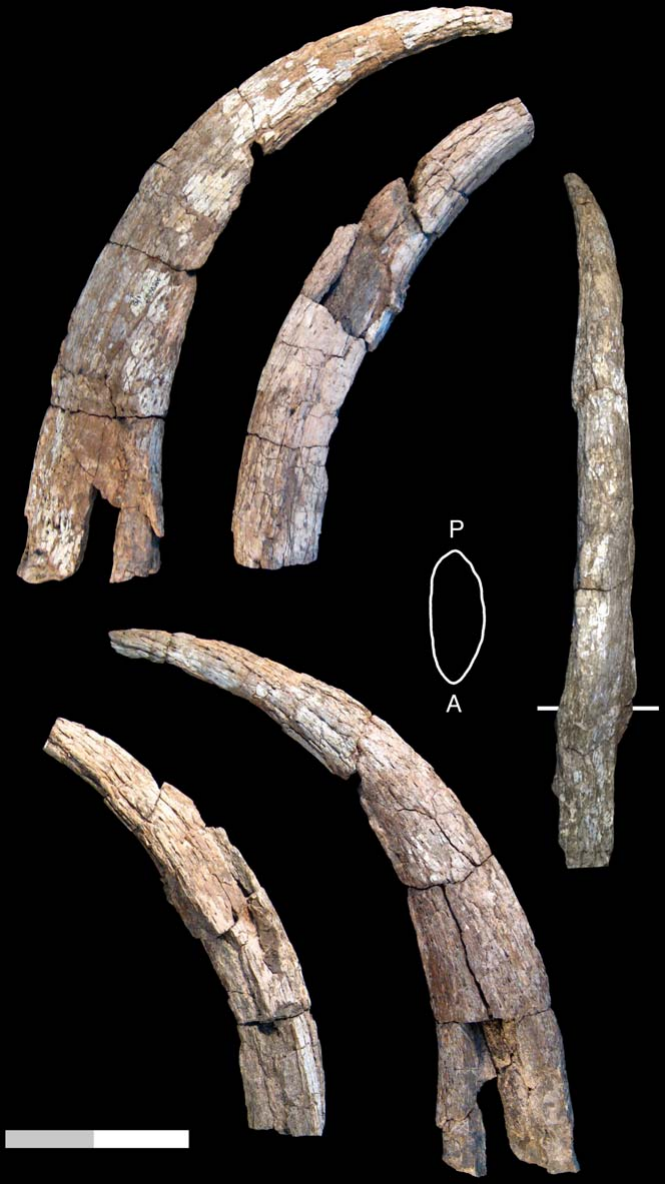
cf. Caprini. AMW-VP-1/51, right and left horn cores. Shown in side views (top and bottom), and the better preserved of the two horn cores in anterior view (right) with sketch of basal cross-sectional shape labelled anterior (A) and posterior (P). Scale bar equals 10 cm total.

AMW-VP-1/51, right and left horn cores (Fig. 5).

#### Age

From the Kuseralee Member of the Sagantole Formation. Fossils come from just below the MA92-15 5.18 Ma basalt, so are estimated to be ≥5.2 Ma [6].

#### Description

AMW-VP-1/51 consists of two horn cores from a single individual, one of which is almost complete and the second more fragmentary (Fig. 5). Given each horn core's great symmetry, it is not evident which is the left and which the right. Horn cores are extremely compressed mediolaterally, with sides flat, and curve posteriorly scimitar-like, lacking torsion. The anterior and posterior surfaces of the horn core are somewhat rounded and not keeled. The cross-section at the base is oval, lacking signs of basal swellings, with the widest transverse diameter located posteriorly. The more complete of the two horn cores preserves what appears to be the horn core base and an attached sliver of pedicel, the internal surface of which appears smooth-walled, indicating the presence of a simple, unstrutted, frontal sinus that reached the pedicel but did not enter the horn core proper. Basal measurements of the more complete horn core are (DTxDAP) 39.3×73.7 mm; approximate complete length along the anterior surface  = 425 mm.

#### Comparisons

In its extreme horn core compression and simple posterior curvature, AMW-VP-1/51 is differentiated from known fossils of Hippotragini of Alcelaphini from the Mio-Pliocene of Africa [18], [21]. The living *Hippotragus niger* often has very strongly compressed horn cores but always retains a rounder horn core base than in AMW-VP-1/51. The taxa most comparable to AMW-VP-1/51 are *Skoufotragus* ( = *Pachytragus) laticeps*, known best from late Miocene Samos (Greece) [22], [23], *Bouria anngettyae* from the 1 Ma Bouri Daka Member (Ethiopia) [24], and *Pachytragus* sp. from the Namurungule Formation (Kenya) [25], [26], [27]. *Skoufotragus laticeps* and *Bouria anngettyae* both resemble the Awash specimen in posterior-curving horn cores that are very mediolaterally flattened, with hollowed pedicels. The degree of posterior curvature in AMW-VP-1/51 is more so than in *S. laticeps*, and more like the condition in *B. anngettyae*. AMW-VP-1/51 differs from *Skoufotragus laticeps* in stronger posterior curvature, larger size, greater medio-lateral compression, and in the lack of asymmetry or swelling at the basal horn core. It differs from *Pachytragus* sp. described from the Namurungule Formation in much the same characters. AMW-VP-1/51 differs from *Bouria anngettyae* in the constancy of the horn core curvature and cross-section, as in *Bouria* the horn core widens disproportionately and asymmetrically at the base.

#### Notes

The subfamily Caprinae traditionally comprises *Pantholops* and three taxonomic groups, Caprini+Rupicaprini+Ovibovini, none of which are likely to be monophyletic [28], [29]. Hassanin and Douzery [30] suggested the use of the name Caprini in place of the traditionally-defined Caprinae, a view more in accord with emerging molecular phylogenetic evidence. I follow these authors' nomenclature, pointing out that Caprini here is synonymous to the Caprinae of many other authors.

The assignment of AMW-VP-1/51 to Caprini is not made with any certainty in large part because the identification of caprins in the Miocene fossil record is not straightforward. For example, the assignment to Caprini of both *Skoufotragus* ( = *Pachytragus*) *laticeps* and *Bouria anngettyae*, the two taxa most comparable to AMW-VP-1/51, is open to consideration. While Gentry [22], [31] argued for the caprin status of *Pachytragus*, Pilgrim “doubtfully” [5] (p.73) and Bosscha-Erdbrink [32] took it to be a hippotragin. Likewise, *Bouria anngettyae* was identified by Vrba [24] as a caprin, but Gilbert [33] noted morphological similarities of this species to the alcelaphin *Parmularius angusticornis*. *Skoufotragus laticeps*, *Bouria anngettyae*, and AMW-VP-1/51 all share strong medio-lateral compression of the horn core and the presence of a smooth-walled frontal sinus extending into the pedicel. Bovids with strong medio-lateral compression of the horn core have often been taken to be caprins, but this character can be present in both hippotragins (e.g. *Hippotragus*) and alcelaphins (e.g. *Parmularius, Damaliscus*). Likewise, the smooth-walled pedicel sinus appears more similar to the condition in Hippotragini and Alcelaphini than to the normally strutted pedicel sinuses of Caprini (*Capricornis* and *Naemorhedus* may be exceptions [34]). Confusion in the assignment of Miocene fossils among Caprini, Hippotragini, and Alcelaphini is not surprising given that these three clades are in fact sister taxa [29]. Certain fossil forms would then be expected to show a mosaic of primitive and derived characters that indicates affinity to the greater clade Caprini+Hippotragini+Alcelaphini, but makes confident assignment to any one of these tribes difficult.

AMW-VP-1/53 is recorded from the KUSM, while two postcranial specimens assigned to cf. Caprini are recorded from the ASKM (STD-VP-2/74, ALA-VP-2/281, Middle Awash database, http://middleawash.berkeley.edu). It is not possible to speculate as to whether the ASKM and KUSM specimens represent the same or different species.

## Discussion

### I. Revision of bovid faunas from 5.6 and 5.2 Ma in the Middle Awash

At 5.77–5.54 Ma and ∼5.2 Ma, the Asa Koma Member (ASKM) and the Kuseralee Member (KUSM) faunas of the Middle Awash, Ethiopia, sample terminal Miocene faunas in eastern Africa [3]. The ASKM and KUSM mammalian faunas show the greatest taxonomic similarity to contemporaneous assemblages from Kenya, Chad, Libya, and the United Arab Emirates, followed by those from Iran, Spain, Greece, and Italy [35]. Assemblages such as the ASKM and KUSM provide a window onto late Miocene biogeographic configurations in Africa, which already had an African-endemic (Ethiopian) character to them but retain a degree of taxonomic continuity with Eurasia, shortly before early Pliocene advent of increased continental endemism (discussed below).

The bovid taxa recorded from the Asa Koma and Kuseralee Members of the Middle Awash are listed in Table 1, updated to reflect the identification of new taxa and revision of specimens presented in this paper. Comparison of the bovid taxa from these two assemblages shows some taxonomic differences between these two assemblages, dated to between 5.77–5.54 Ma and just older than 5.2 Ma, respectively [6], [16]. About half of the bovid taxa in each member are not found in the other. At minimum, four out of ten earlier ASKM bovid species are not represented in the later KUSM (*Tragoportax* sp. ‘large’, *Ugandax* sp., *Prostrepsiceros* cf. *vinayaki*, *Zephyreduncinus oundagaisus*). Seven out of thirteen KUSM bovids are not recorded from the earlier ASKM (*Ugandax demissum, Aepyceros* cf. *premelampus*, *Gazella* sp., three reduncins, and a hippotragin). None of these taxonomic differences are the result of clear examples of phyletic evolution, as found in other parts of the total assemblage comparison by Haile-Selassie et al. [36], but some may be explained by sampling biases probably related to the local absence of appropriate habitats. *Ugandax* sp. from the AKSM is not a likely ancestor for *U. demissum* (reassigned from *Simatherium* following Gentry [37]) from the KUSM, and the similarity of the former to related species from the Pliocene [37], [38] suggests its absence from the KUSM is likely an artifact of sampling. Similarly, Hippotragini, *Gazella*, and *Aepyceros* are recorded from sites older than the KUSM (e.g. Lower Nawata) [39], meaning their absence from the ASKM is also likely to be the result of sampling bias. The taxonomic differences that remain significant are summarized by the presence of *Zephyreduncinus oundagaisus* and *Prostrepsiceros* cf. *vinayaki* in the ASKM, and three different reduncins and *Ugandax demissum* in the KUSM. Small sample sizes (Table 1), however, limit any confident interpretation for the differences. Overall, the bovid fauna does not provide strong evidence for any major evolutionary turnover taking place in the time between the two assemblages [36]. The same record, however, does leave open the possibility of some degree of paleoenvironmental differences between the two members.

**Table 1:** Revised faunal lists for fossil Bovidae of the Asa Koma (5.77–5.54 Ma) and Kuseralee (∼5.2 Ma) members of the Middle Awash.

**ASKM**	**KUSM**
cf Tragoportacini (6)	
Tragoportacini indet. (4)	
*Tragoportax* cf. *abyssinicus* (4)	*Tragoportax abyssinicus* (2)
*Tragoportax* sp. ‘large’ (11)	
cf. Bovini (4)	
Bovini indet. (10)	Bovini indet. (18)
*Ugandax* sp. (2)	
	*Ugandax demissum* (1)
cf. Tragelaphini (7)	cf. Tragelaphini (12)
*Tragelaphus moroitu* (10)	*Tragelaphus moroitu* (25)
	*Aepyceros* cf. *premelampus* (4)
*Prostrepsiceros* cf. *vinayaki* (2)	
	*Gazella* sp. (5)
*Madoqua* sp. (2)	*Madoqua* sp. (1)
*Raphicerus* sp. (1)	*Raphicerus* sp. (2)
Reduncini gen et sp indet (17+)	Reduncini gen et sp indet (15+)
*Kobus* cf. *porrecticornis* (13)	*Kobus* cf. *porrecticornis* (1)
*Zephyreduncinus oundagaisus* (7)	
	*Redunca ambae* (4)
	*Kobus* aff. *oricornis* (3)
	*Kobus* cf. *basilcookei* (2)
	Hippotragini indet. (7)
cf. Caprini (2)	cf. Caprini (1)
**Total NISP** 102+	103+

Number in parentheses is the number of identified specimens (NISP), with counts compiled from Haile-Selassie et al. [20], [36], the Middle Awash online database (http://middleawash.berkeley.edu), Vrba [87], and this study.

The identification of *Prostrepsiceros* cf. *vinayaki* from the ASKM indicates biogeographic linkages with the Baynunah and the Siwaliks, but does not significantly alter Bernor et al. 's [35] biogeographic analysis of the Middle Awash Asa Koma fauna, which found the greatest overall resemblance to that of the Lothagam Nawata Formation. Rather, *Prostrepsiceros* cf. *vinayaki* highlights the presence of Eurasian elements in late Miocene eastern Africa while also demonstrating the relatively restricted nature of Eurasian-African faunal exchanges compared to faunal dispersion within Africa itself.

### II. Review of African Fossil Bovids of Eurasian Affinities since 8 Ma by Tribe

Bovids are a widespread and diverse group ideally suited for biogeographic studies. Modern bovid ranges conform almost perfectly to Wallace's biogeographic zonations: though there are some 132 extant bovid species, practically none of these possesses a geographic range that significantly traverses any of the traditionally-defined biogeographic boundaries. Perhaps the only exception might be produced if the three ibex species (*Capra ibex, C. sibirica, C. nubiana*) were considered collectively [40], providing a range that covers parts of Africa, southern Europe, and Central Asia. In an attempt to better understand the history of development of the Ethiopian realm, I here review the record of sub-Saharan African fossil bovids with ranges significantly traversing the boundaries of modern biogeographic realms, focusing mainly on the record of the last 8myr.

#### Antilopini

Besides *Prostrepsiceros* cf. *vinayaki* described above, perhaps the only other occurrence of a sub-Saharan fossil antilopin of Eurasian affinity is that of *Antilope* aff. *subtorta* from the late Pliocene Member C of the Shungura Formation [41]. *Antilope* is otherwise represented in the Pleistocene Pinjor Formation of the Siwaliks by *Antilope subtorta* [5], [42] and today in the Indian subcontinent by the living *Antilope cervicapra*.

From the ca.9.5 Ma [43] Namurungule Formation in Kenya, Nakaya [44] reported ‘*Ouzocerus?* sp.’ (previously *Palaeoreas* sp. [25], [26]), an antilopin otherwise recorded from Greece [45] and Tunisia (Thomas in [44]). An updated listing of the Namurungule fauna, however, omits *Ouzocerus* [27].

#### Caprini

The new Middle Awash cf. Caprini specimen joins a list of caprin or caprin-like taxa known from isolated occurrences in the sub-Saharan African late Neogene record. These include *Pachytragus* sp. from the Namurungule Formation [26], [27], [44], *Budorcas churcheri* from Hadar [46], Ovibovini indet. from Langebaanweg [47], *Bouria anngettyae* and *Nitidarcus asfawi* from the Middle Awash Pleistocene [24], *Makapania broomi* and related species from late Pliocene to Holocene sites of South Africa [48], [49], [50], and numerous records of Caprini gen. et sp. indet. from Turkana Basin sites aged 3–1 Ma [41], [51], [52], [53]. To this list I would also add *Brabovus nanincisus* from Laetoli, originally assigned to Bovini [54]. *Brabovus nanincisus* bears characteristics that preclude inclusion in Bovini, or even Bovinae, including horn cores lacking keels and with a prominent raised lip at horn core–pedicel border; a deep postcornual fossa; a rounded braincase that is wider anteriorly, with laterally-facing occipital surfaces, mastoids located fairly far anterior on the skull, and a flexed and ventrally extended basicranium; a lower p2 that is relatively reduced in size and morphology; and a dual infraorbital foramen [55]. Vrba and Gatesy [56] ruled out *Brabovus nanincisus* from being a hippotragin. I propose that the presence of the above-mentioned traits rule out the possibility of this species being a bovin, and, along with the strutted frontal sinuses and small central incisor, favor the placement of *Brabovus nanincisus* in Caprini.

The biogeographic implications of the African caprin fossil record are not immediately evident. Vrba [53] interpreted the patchy fossil record of Caprini in Africa to represent repeated episodes of faunal immigration into the continent from Eurasia, coincident with episodes of global cooling and the opening of land bridge connections (her “traffic-light” model). This hypothesis might find support in the records of *Pachytragus*, *Budorcas*, and *Makapania*, taxa with demonstrated affinities to Eurasian clades, but less so by the large number of taxonomically indeterminate caprin fossils, or even *Bouria, Nitidarcus,* and *Brabovus,* that have not been associated phylogenetically with any Eurasian caprin clades. Without further information, the majority of the sub-Saharan African caprin fossil record might just as well be sampling endemic African caprin lineages. Given an ecological preferences for mountainous terrain, their rarity in rift-axial fluvial fossil deposits would not be a surprise.

#### Reduncini

Reduncin antelopes are today restricted to sub-Saharan Africa, though fossil reduncins are also recorded from North Africa, the Levant, and the Indian subcontinent (and doubtfully from Iran and Spain [53]). The fossil record of Reduncini from the Siwaliks is extensive, spanning the late Miocene to the Pleistocene (Dhok Pathan to Pinjor formations) and comprising diverse species [5], [53]. The reduncin *Kobus* ( = *Dorcadoxa*) *porrecticornis* was first described by Pilgrim [5] from the Dhok Pathan deposits of the Siwaliks (recorded from 9.3 to 8.0 Ma) [11]. *Kobus porrecticornis* (or *K.* cf. *porrecticornis*) has since been recorded from both the Middle Awash ASKM and the KUSM, and also from Mpesida, Lukeino, Baard's Quarry at Langebaanweg, and the Upper Nawata [20], [39], [47], [57]. These sites all date to between ∼6.5 and 5 Ma, including presumably the specimens from Baard's Quarry, which is a mixed assemblage [58]. Other reducins are known from the Pliocene and Pleistocene Tatrot and Pinjor Formations [5]. Though their relationships to contemporaneous African reduncins are not clear, there is some indication that Siwaliks Plio-Pleistocene reduncins evolved from the Dhok Pathan taxa without significant connection with African species [53], [59].

Though providing the necessary route between Africa and the Indian subcontinent, no fossil reduncins are recorded from the Arabian Peninsula (Fig. 4), neither from the Baynunah Formation [8], nor the Pleistocene of Nafud [60]. Despite sample sizes being small, this absence remains intriguing, particularly since reduncins are recorded from relatively similar faunas and paleoenvironments at Toros-Menalla and Sahabi, being particularly abundant at the former site [18], [61]. The absence of Reduncini among Arabian fossil faunas may reflect the absence of appropriate habitats to sustain these antelopes in the Peninsula for significant periods of geological time. Evidence for arid conditions contemporaneous with the Baynunah river system [62] suggests that the Arabian Peninsula has been characterized by aridity since at least the late Miocene, with climatic variations continuously acting to shift the availability and distribution of limited freshwater habitats. Assuming they had the ecological preferences of their modern counterparts, reduncins such as *Kobus porrecticornis* would have required permanent wetlands and watered habitats, and only managed intermittent passage through, but not long-term persistence in, the Arabian Peninsula. Perhaps a modern analog may be sought in the Nile River, which supports several reduncin species along its upper reaches in Uganda and Sudan, but none as it traverses the Egyptian Sahara. In contrast, antilopins such as *Prostrepsiceros vinayaki* may have been sufficiently adapted to semi-arid conditions to persist and mark their presence in the late Miocene Arabian fossil record.

#### Tragoportacini (‘Boselaphini’)

**Figure 6:**
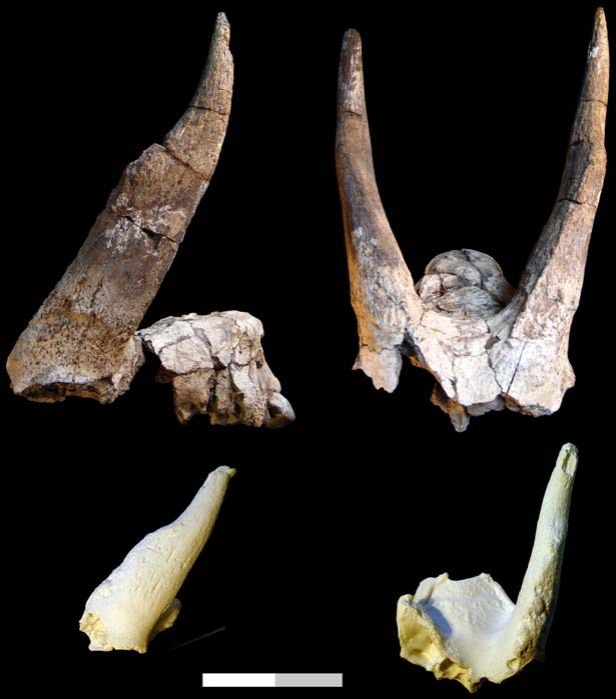
Type specimens of *Tragoportax abyssinicus* (AMH-VP-1/1, top) and *Miotragocerus monacensis* (cast in Natural History Museum, London, bottom) in left lateral and anterior views, showing the relative similarities between these two taxa. Scale bar equals 10 cm total.

Tragoportacini [63], including primarily species of *Tragoportax* and *Miotragocerus*, is well represented throughout the late Miocene of Europe and Asia, but is poorly known from Africa. Recent years have seen the documentation of several tragoportacin taxa from late Miocene African sites, and I here note similarities between these and certain Eurasian tragoportacins. *Tragoportax abyssinicus* was named by Haile-Selassie et al. [20] from the Kuseralee Member (and might be represented in the ASKM by *T.* cf. *abyssinicus*). Similar bovids to *T. abyssinicus* from the African fossil record are *Tragoportax* sp. ‘A’ from the Lower and Upper Nawata members at Lothagam [39], and to a lesser extent *Tragoportax* sp. ‘B’ from the same deposits. The above-named taxa all share small size and straight, upright horns often marked by a prominent anterior demarcation and anterior-ward recurvature towards the tips. However, further comparisons suggest to me that *T. abyssinicus* finds its closest morphological match with *Miotragocerus monacensis*, represented by the syntype calvarium from Oberföhring, near Munich [64]. I have examined a cast of this at the Natural History Museum (London), and the differences between the Awash specimens and *T. monacensis* come down to a few basic characters. *T. abyssinicus* differs from *M. monacensis* in larger size, horn cores that are relatively longer and more quadrangular in cross-section, and in the presence of a raised ridge between the horn cores. Otherwise the two taxa are similar (Fig. 6), and one would probably be justified reassigning *Tragoportax abyssinicus* to *Miotragocerus*. I hesitate to do this here, however, given continuing uncertainty on how to diagnose and differentiate *Tragoportax* and *Miotragocerus*, and the resulting unclear benefits of such a generic distinction. From the Siwaliks, *Sivaceros vedicus* is also a close match for *T. abyssinicus*, particularly the horn core of *S.* cf. *vedicus* illustrated by Pilgrim [5]. Likewise, *Tragoportax* sp. ‘A’ from Lothagam [39] also also compares well to *Sivaceros vedicus*.

Other *Tragoportax* species are represented in Africa by *Tragoportax cyrenaicus* (originally *Miotragocerus cyrenaicus*) from Sahabi [65], *T.* aff. *cyrenaicus* from Lower Nawata, Upper Nawata, and Apak members at Lothagam [39], *Tragoportax* sp. ‘large’ from the Asa Koma Member [20], *T. acrae* (originally *Mesembriportax acrae*) from Langebaanweg [47], and a calvarium referred to *Tragoportax* sp. from the Namurungule Formation [27]. *Tragoportax cyrenaicus* is only otherwise recorded from the Baynunah Formation [8] while *T. acrae* is known only from its type locality.

**Figure 7:**
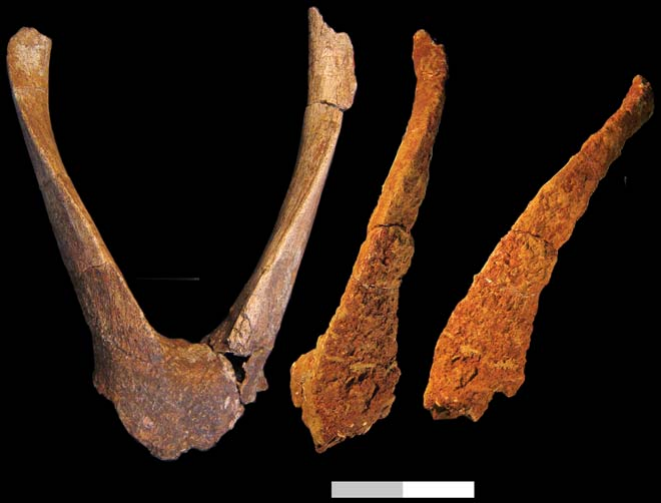
*Tragoportax* aff. *cyrenaicus* (KNM-LT 23149) in anterior view and AMNH 101260, a left horn core, in anterior and lateral views. From Lothagam and the Siwaliks, respectively, these two specimens show great morphological similarities and call for further comparison of African and Asian Tragoportacini. Scale bar equals 10 cm total.

The Lothagam *Tragoportax* aff. *cyrenaicus*, while close to the Baynunah and Sahabi *Tragoportax*, also merits comparison to Siwaliks forms (Fig. 7). KNM-LT 23149, a partial frontal with left and right horn cores from the Lower Nawata, finds a good match in AMNH 101260, a left horn core from the Dhok Pathan deposits of the Siwaliks labelled as *T. punjabicus* (junior synonym of *T. rugosifrons* [66] or senior synonym of *T. browni* and *T. curvicornis* [23], [67]). In AMNH 101260 and KNM-LT 23149, the horn cores lack significant posterior curvature and possess marked torsion of about 90° total, such that the medial surface of the horn core clearly comes to face anteriorly in its distal sections. These characters are absent in other AMNH material of *T. rugosifrons*, *T. browni,* and *T. curvicornis* that I was able to see, and call for further comparison of African and Siwaliks tragoportacins.

The high taxonomic and morphological diversity that exists within and among species of *Tragoportax* and *Miotragocerus* means it is difficult to interpret with any confidence the similarities mentioned above between certain African and Eurasian tragoportacins. Further work is needed to adequately reconstruct the phylogeny of tragoportacins from different assemblages in Asia, Europe, and Africa. For the moment, it is sufficient to point out that the similarity of African tragoportacins to specific tragoportacins from Europe and southern Asia provides evidence for a greater degree of biotic continuity between Africa and Eurasia during the late Miocene than is found later in time. This echoes the record presented by *Prostrepsiceros vinayaki* and *Kobus porrecticornis*. Unlike antilopins and reduncins, tragoportacins experience a global extinction in the earliest Pliocene, vanishing completely and mysteriously from the European, Asian, and African records at around or just after 5 Ma.

#### Alcelaphini, Hippotragini, “Neotragini.”

One notable occurrence of bovid taxa of possibly African origin in late Miocene Europe comes from the early late Miocene Grosseto lignites of Tuscany. These include the alcelaphin-like *Maremmia haupti,* and *Tyrrhenotragus gracillimus*, a “neotragin” [68].

Otherwise, Alcelaphini, along with Hippotragini, are restricted for the duration of their histories to Africa and Arabia, with the exception of short-lived dispersals to the Indian subcontinent between around 3 Ma and 2.5 Ma [53]. Though today extinct in North Africa and the Levant, *Alcelaphus buselaphus* is recorded from late Pleistocene sites in the Levant, and was widespread in North Africa until the early 20th Century [69], [70], [71].

### III. Development of the Ethiopian Biogeographic Realm as evidenced by the Bovid Fossil Record

**Figure 8:**
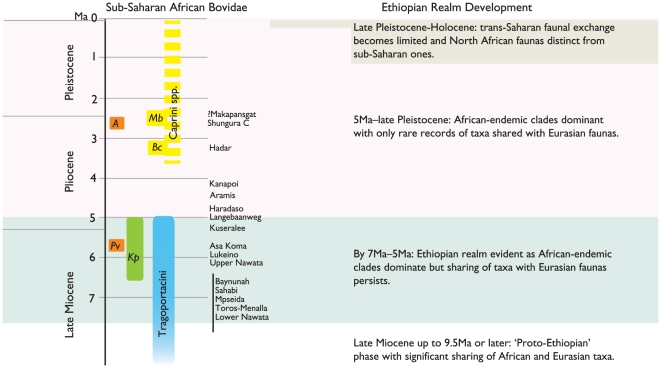
A summary of the record of sub-Saharan fossil bovids indicating faunal exchange with Eurasia, and the corresponding history of the Ethiopian biogeographic realm. The Plio-Pleistocene record of African caprins is spotty, and it is not evident whether the majority of its taxa represent Eurasian immigrants. Abbreviations: *A*, *Antilope* aff. *subtorta*; *Bc*, *Budorcas churcheri*; *Kp*, *Kobus porrecticornis*; *Mb*, *Makapania broomi; Pv*, *Prostrepsiceros* cf. *vinayaki*.

Thomas [40], [72], following Gentry [73], discussed the development of African biogeography through the bovid fossil record of the middle Miocene and the early late Miocene, indicating significant Eurasian influences and the appearance of what he termed a ‘proto-Ethiopian’ phase during this time. The Namurungule fauna, dated to around 9.5 Ma [27], bears strong Eurasian affinities and can also be included in the early late Miocene proto-Ethiopian phase. I here outline three further phases in the development of modern African biogeography since the latter half of the late Miocene, or from around 7 Ma (Fig. 8). These three phases are also generally recognizable in other aspects of the African large mammal fossil record as well (though the micromammalian record often offers a different view [17], [74]). The first two phases are based on the review presented in this work, while the third phase is based mainly on the recent work of Geraads [74].

I. By 7 Ma and up to around 5 Ma: The main faunal characteristics of the Ethiopian biogeographic realm are by this time already developed and recognizable. The Ethiopian realm covers the entirety of Africa and Arabia, with relatively porous boundaries permitting exchange with Eurasian faunas.

During this time all over Africa, fossil bovid tribal makeup is characteristically African in nature, but taxa common to Eurasia, namely the Tragoportacini, are present and diverse. Thomas [40: 251] noted that the “emergence of the true Ethiopian fauna” had occurred by 7 Ma. This is reflected in that, by this time, African bovid faunas were dominated by newly emerged African tribes (Tragelaphini, Alcelaphini, Hippotragini, Aepycerotini) and lineages (of Reduncini and Bovini). However, while not abundant, Tragoportacini remain present and ubiquitous in Africa, a strong reminder of faunal continuity with much of Eurasia. Similarly, the wide ranges of *Kobus porrecticornis* and *Prostrepsiceros vinayaki* provide further evidence for cross-continental faunal exchange. Additional records of admixture of African and Eurasian faunas during this time come from Spain [75], Italy [76], Libya [77], and the Arabian Peninsula [8]. Faunal exchange between sub-Saharan Africa, North Africa, and Arabia at this time is fluid [17], [74], [77], [78], and the balance of taxa unites these regions under a single Ethiopian biogeographic realm. Regional bioprovinciality is however present, as evidenced by the Chado-Libyan biogeographical province that united the Lake Chad Basin to Sirt Basin in Libya [61], [79].

II. ∼5 Ma to the late Pleistocene: The Ethiopian realm continues to cover the entirety of Africa and Arabia, though barriers to faunal exchange with Eurasia are now significant.

Among bovids at this time, there is an almost total dominance of tribes and lineages of African origin, with only rare occurrences of taxa shared with any Eurasian sites. The extinction of Tragoportacini around 5 Ma leaves African tribes (Tragelaphini, Alcelaphini, Hippotragini, Aepycerotini, Cephalophini) and lineages (of Bovini, Reduncini, and Antilopini) in near total faunal dominance. Sub-Saharan African fossil assemblages such as those of the Haradaso Member of the Sagantole Formation at about 4.9 Ma (personal observations), Lower Aramis Member at 4.4 Ma [80], [81] or Kanapoi at 4.1 Ma [82] comprise bovid faunas dominated by species and clades of African origin. A similar pattern of post-Miocene endemism has been documented among Carnivora [83]. During the late Pliocene and Pleistocene, any indications among Bovidae of Eurasian influence into sub-Saharan Africa are almost entirely restricted to rare records of Caprini (e.g. *Budorcas churcheri*, *Makapania broomi*), and the Shungura *Antilope*. The short-lived appearance of some alcelaphins and hippotragins in the Indian subcontinent in the late Pliocene provides evidence for dispersals out of Africa. The carnivore fossil record also provides evidence for greater migration out of Africa than into Africa at this time [84]. North African faunas from this time continue to show a dominance of African, rather than Eurasian, taxa [85], [86].

III. Late Pleistocene to Recent: The northern limit of the Ethiopian realm shifts south towards its present configuration about the Tropic of Cancer.

In North Africa, the immigration of Palaearctic taxa and the loss of faunal elements in common with sub-Saharan Africa changes the makeup and continental affinities of this region's fauna [74]. This leads to the modern-day classification of North Africa in the Palaearctic realm and the redrawing of the Palaearctic-Ethiopian realm boundary along the Saharo-Arabian desert belt. Records of Eurasian bovids in sub-Saharan Africa are absent or rare. Occasional dispersals of bovids and other large mammals from sub-Saharan African into North Africa and the Levant take place into the late Holocene [69].

### Conclusions

The Ethiopian biogeographic realm appears to have had a distinct history of assembly through the Neogene, reflecting a pattern of ever-increasing isolation of African faunas since the late Miocene. The ‘isolating barriers’ of Wallace, defining the northern boundary of the Ethiopian realm, were in place by 7 Ma, though the geographic location of this boundary, and its permeability to African and Eurasian taxa, has changed over time. The presence of *Prostrepsiceros* cf. *vinayaki* and a possible caprin in the ASKM and KUSM, taken with the remainder of the bovid record reviewed above, highlights a greater rate of interchange of faunal elements between Eurasia and Africa in the late Miocene and up to around 5 Ma, than found later in time. The majority of the African Pliocene and Pleistocene record is exceptionally poor in Eurasian elements and an African-endemic fauna dominates. Of interest at this time is what appears to be a greater rate of faunal dispersal out of Africa than into it, documented also among other taxa. Throughout most of the last 7myr, the Ethiopian realm covered the entirety of Africa and Arabia. The distinction of North Africa from Sub-Saharan Africa, and the delineation of the northern limits of the modern Ethiopian realm along the Saharo-Arabian desert belt, would come only in the late Pleistocene, presumably on account of increased sub-tropical aridification.

## Materials and Methods

Fossils were studied in collections housed at the National Museum of Ethiopia, National Museums of Kenya, the American Museum of Natural History, the Abu Dhabi Authority for Culture and Heritage, and the Natural History Museum, London. Fossil specimens were measured using digital calipers, angle measure, and metric tape.
